# Clinical outcomes in individuals at clinical high risk of psychosis who do not transition to psychosis: a meta-analysis

**DOI:** 10.1017/S2045796021000639

**Published:** 2022-01-19

**Authors:** Gonzalo Salazar de Pablo, Livia Soardo, Anna Cabras, Joana Pereira, Simi Kaur, Filippo Besana, Vincenzo Arienti, Francesco Coronelli, Jae Il Shin, Marco Solmi, Natalia Petros, Andre F. Carvalho, Philip McGuire, Paolo Fusar-Poli

**Affiliations:** 1 Early Psychosis: Interventions and Clinical-detection (EPIC) Lab, Department of Psychosis Studies, Institute of Psychiatry, Psychology & Neuroscience, King's College London, London, UK; 2 Department of Child and Adolescent Psychiatry, Institute of Psychiatry and Mental Health, Hospital General Universitario Gregorio Marañón School of Medicine, Universidad Complutense, Instituto de Investigación Sanitaria Gregorio Marañón (IiSGM), CIBERSAM, Madrid, Spain; 3 Child and Adolescent Mental Health Services, South London and Maudsley NHS Foundation Trust, London, UK; 4 Department of Child and Adolescent Psychiatry, Institute of Psychiatry, Psychology and Neuroscience, King's College London, London, UK; 5 Department of Brain and Behavioral Sciences, University of Pavia, Pavia, Italy; 6 Department of Neurology and Psychiatry, University of Rome La Sapienza, Rome, Italy; 7 Lisbon Psychiatric Hospital Center, Lisbon, Portugal; 8 Department of Psychosis Studies, Institute of Psychiatry, Psychology and Neuroscience, King's College London, London, UK; 9 Department of Paediatrics, Yonsei University College of Medicine, Seoul, Republic of Korea; 10 Department of Psychiatry, University of Ottawa, Ontario, Canada; 11 Department of Mental Health, The Ottawa Hospital, Ontario, Canada; 12 Ottawa Hospital Research Institute (OHRI) Clinical Epidemiology Program University of Ottawa, Ottawa, Ontario; 13 IMPACT (Innovation in Mental and Physical Health and Clinical Treatment) Strategic Research Centre, School of Medicine, Barwon Health, Deakin University, Geelong, VIC, Australia; 14 OASIS service, South London and Maudsley NHS Foundation Trust, London, UK; 15 National Institute for Health Research, Maudsley Biomedical Research Centre, South London and Maudsley NHS Foundation Trust, London, UK

**Keywords:** Psychosis, clinical high risk, clinical outcomes, progression, transition, meta-analysis

## Abstract

**Aims:**

The clinical outcomes of individuals at clinical high risk of psychosis (CHR-P) who do not transition to psychosis are heterogeneous and inconsistently reported. We aimed to comprehensively evaluate longitudinally a wide range of outcomes in CHR-P individuals not developing psychosis.

**Methods:**

“Preferred Reporting Items for Systematic reviews and Meta-Analyses” and “Meta-analysis Of Observational Studies in Epidemiology”-compliant meta-analysis (PROSPERO: CRD42021229212) searching original CHR-P longitudinal studies in PubMed and Web of Science databases up to 01/11/2021. As primary analysis, we evaluated the following outcomes within CHR-P non-transitioning individuals: (a) change in the severity of attenuated psychotic symptoms (Hedge's *g*); (b) change in the severity of negative psychotic symptoms (Hedge's *g*); (c) change in the severity of depressive symptoms (Hedge's *g*); (d) change in the level of functioning (Hedge's *g*); (e) frequency of remission (at follow-up). As a secondary analysis, we compared these outcomes in those CHR-P individuals who did not transition *vs.* those who did transition to psychosis at follow-up. We conducted random-effects model meta-analyses, sensitivity analyses, heterogeneity analyses, meta-regressions and publication bias assessment. The risk of bias was assessed using a modified version of the Newcastle-Ottawa Scale (NOS).

**Results:**

Twenty-eight studies were included (2756 CHR-P individuals, mean age = 20.4, 45.5% females). The mean duration of follow-up of the included studies was of 30.7 months. Primary analysis: attenuated psychotic symptoms [Hedges’ *g* = 1.410, 95% confidence interval (CI) 1.002–1.818]; negative psychotic symptoms (Hedges’ *g* = 0.683, 95% CI 0.371–0.995); depressive symptoms (Hedges’ *g* = 0.844, 95% CI 0.371–1.317); and functioning (Hedges’ *g* = 0.776, 95% CI 0.463–1.089) improved in CHR-P non-transitioning individuals; 48.7% remitted at follow-up (95% CI 39.3–58.2%). Secondary analysis: attenuated psychotic symptoms (Hedges’ *g* = 0.706, 95% CI 0.091–1.322) and functioning (Hedges’ *g* = 0.623, 95% CI 0.375–0.871) improved in CHR-P individuals not-transitioning compared to those transitioning to psychosis, but there were no differences in negative or depressive symptoms or frequency of remission (*p* > 0.05). Older age was associated with higher improvements of attenuated psychotic symptoms (*β* = 0.225, *p* = 0.012); publication years were associated with a higher improvement of functioning (*β* = −0.124, *p* = 0.0026); a lower proportion of Brief Limited Intermittent Psychotic Symptoms was associated with higher frequencies of remission (*β* = −0.054, *p* = 0.0085). There was no metaregression impact for study continent, the psychometric instrument used, the quality of the study or proportion of females. The NOS scores were 4.4 ± 0.9, ranging from 3 to 6, revealing the moderate quality of the included studies.

**Conclusions:**

Clinical outcomes improve in CHR-P individuals not transitioning to psychosis but only less than half remit over time. Sustained clinical attention should be provided in the longer term to monitor these outcomes.

## Introduction

Indicated prevention in individuals at clinical high risk for psychosis (CHR-P) is one of the most promising primary preventive approaches in psychiatry (Fusar-Poli *et al*., 2017*b*). CHR-P individuals are young and they accumulate risk factors such as living alone or being unemployed (Fusar-Poli *et al*., 2017*e*; Radua *et al*., 2018; Oliver *et al*., 2019) that enrich their level of risk for psychosis (Fusar-Poli *et al*., 2016*c*). In turn, this can lead to functional impairments (Fusar-Poli *et al*., 2015*c*) and the onset of attenuated psychotic symptoms (Fusar-Poli *et al*., 2017*c*). The distress associated with these experiences can prompt CHR-P individuals to seek help (Falkenberg *et al*., 2015) at specialised mental health clinics (Kotlicka-Antczak *et al*., 2020; Salazar de Pablo *et al*., 2021*a*). In these clinics, the prognosis is formulated reaching very good accuracy using psychometric instruments (Fusar-Poli *et al*., 2015*a*).

The majority of CHR-P individuals do not transition to psychosis within the first 2 years of presentation. After 2 years, 16% of CHR-P individuals transition to psychosis, and the transition risk continues to rise until about 4 years of follow-up, reaching 36% at 10–11 years (Salazar de Pablo *et al*., 2021*c*). Clinical outcomes in CHR-P individuals who do not transition to psychosis remain scattered, heterogeneous and inconsistent (Simon *et al*., 2011). It remains unclear how many will improve, permanently or only temporarily (Schultze-Lutter, 2009; Fusar-Poli *et al*., 2013), or whether the poor mental health outcomes observed are mostly driven by the presence of transitioning CHR-P individuals. Longitudinal research comparing individuals who develop psychosis with those who do not is overall inconsistent (Fusar-Poli *et al*., 2013).

Following an earlier meta-analysis, published 10 years ago, evaluating the proportion of individuals who do not transition to psychosis and the frequency of remission (Simon *et al*., 2011), a more recent update found that 28–71% of CHR-P individuals who do not transition to psychosis, do not remit from their CHR-P either and 22–82% still have one or more clinical diagnosis in the long-term (Beck *et al*., 2019*a*). Additional meta-analyses have focused on other clinical outcomes in all CHR-P individuals (transitioning and non-transitioning individuals), including the level of functioning (Fusar-Poli *et al*., 2015*c*, 2017*e*), quality of life (Fusar-Poli *et al*., 2015*c*), comorbid disorders (Albert *et al*., 2018) and remission (Simon *et al*., 2013). However, no comprehensive meta-analysis has addressed these outcomes together in individuals at CHR-P not developing psychosis. Also, no meta-analysis has evaluated outcomes in CHR-P individuals not transitioning to psychosis, comparing to those developing it. We aimed to comprehensively assess the broad longitudinal clinical outcomes of attenuated psychotic symptoms, negative symptoms, depressive symptoms, functioning and remission in CHR-P individuals who did not transition to psychosis. Furthermore, we aimed to compare these outcomes between those CHR-P transitioning or not to psychosis, while controlling for some potential moderators.

## Methods

The protocol for this study was registered on PROSPERO (CRD42021229212). This study was conducted in accordance with the ‘Preferred Reporting Items for Systematic reviews and Meta-Analyses’ (PRISMA) (Moher *et al*., 2009) (online Supplementary eTable 1), ‘Meta-analysis Of Observational Studies in Epidemiology’ (MOOSE) (Stroup *et al*., 2000) (online Supplementary eTable 2) and ‘Reporting Tool for Practice Guidelines in Health Care’ (RIGHT) (Chen *et al*., 2017) statements.

### Literature search

A multi-step literature search from inception until 1 November 2020 was performed by independent researchers on PubMed and on the Web of Science database (Clarivate Analytics), which incorporates the Web of Science Core Collection, MEDLINE, BIOSIS Citation Index, KCI-Korean Journal Database, Russian Science Citation Index and SciELO Citation Index.

The following search terms were applied: ‘risk’ OR ‘prodrom*’ OR ‘prediction’ OR ‘onset’ OR ‘ultra-high risk’ OR ‘clinical high risk’ OR ‘attenuat*’ OR ‘APS’ OR ‘high risk’ OR ‘BLIPS’ OR ‘brief limited’ OR ‘brief intermitent’ OR ‘genetic high risk’ OR ‘GRD’ OR ‘at risk mental state’ OR ‘risk of progression’ OR ‘progression to first-episode’ OR ‘basic symptoms’ AND ‘psychosis’ OR ‘schizophrenia’ OR ‘schizoaffective’. We manually reviewed the references of previously published meta-analyses and extracted additional relevant titles. Articles identified were reviewed as abstracts. The full texts of the relevant manuscripts were assessed for eligibility. After the exclusion of those that did not meet our inclusion criteria, final decisions were made regarding their inclusion in the meta-analysis. Disagreements in selection criteria were resolved through discussion and consensus.

### Condition and individuals being studied

Studies included were (a) original articles; (b) conducted on CHR-P individuals according to established psychometric instruments (online Supplementary eMethods 1); (c) conducted on CHR-P individuals who did not transition to psychosis (compared or not with those transitioning to psychosis, online Supplementary eMethods 1); (d) cohort studies that provided longitudinal (baseline and follow-up data) (see online Supplementary eMethods 2); (e) published in English.

Studies excluded were (a) review papers, clinical case studies, conference proceedings, study protocols or grey literature; (b) studies conducted on individuals not formally assessed for CHR-P criteria, including those with a schizotypal personality disorder or those with a genetic risk for psychosis (twins, first- or second-degree relatives) without impaired functioning; (c) studies in which transition status at follow-up was not reported; (d) cross-sectional studies; (e) studies in another language other than English; (f) overlapping studies. When a study included data on both individuals who transitioned to psychosis and those who did not, the study was only included if it provided independent stratified outcome results for the two groups of participants. Randomised controlled trials were included using only the placebo/needs-based intervention arm, if available. When there were two or more studies from the same centre, we contacted the authors to clarify whether there was an overlap in the respective samples and the largest and most recent sample was retained.

### Outcomes

Outcomes measured were: (a) change (baseline to follow-up) in the severity of attenuated psychotic symptoms; (b) change (baseline to follow-up) in the severity of negative symptoms; (c) change (baseline to follow-up) in the severity of depressive symptoms; (d) change (baseline to follow-up) in the level of functioning; (e) frequency of remission (at follow-up). These outcomes were operationalised as indicated in online Supplementary eTable 3.

### Data extraction and descriptive variables

Independent researchers extracted data from all the included studies into a database. The data were then cross-checked by a third researcher to ensure high quality of data extraction. Descriptive variables included the following information (more details can be found in online Supplementary eMethods 2): first author and year of publication; country; design; CHR-P sample size; CHR-P subgroups; age; sex; CHR-P assessment tools (see online Supplementary eMethods 1); follow up period; outcome data (at baseline and at follow-up); duration of untreated attenuated psychotic symptoms; transition status; International Classification of Diseases (ICD)-defined (World Health Organization, 2018) or Diagnostic and Statistical Manual of Mental Disorders (DSM)-defined (American Psychiatric Association, 2013) comorbidity; exposure to baseline interventions. For each outcome, data (severity or levels for outcomes a-d and raw counts for outcome e) were extracted at baseline and at 12 months (6–17.9 months); 24 months (18–35.9 months); ⩾36 months follow-up. These outcome data were extracted for both individuals who did not transition and those who did transition to psychosis.

### Risk of bias (quality) assessment

The risk of bias was assessed using a modified version of the Newcastle-Ottawa Scale (NOS) for cohort studies. Studies were awarded 0–8 points according to their representativeness, exposure, outcomes, follow-up period and losses to follow-up (online Supplementary eTable 4).

### Strategy for data synthesis

The primary analysis focused on outcomes within CHR-P individuals who did not transition to psychosis: (a) change (baseline to follow-up) in the severity of attenuated psychotic symptoms; (b) change (baseline to follow-up) in the severity of negative symptoms; (c) change (baseline to follow-up) in the severity of depressive symptoms; (d) change (baseline to follow-up) in the level of functioning, (e) frequency of remission (at follow-up). Outcomes a-d were estimated using the Hedges’ *g* (Hedges, 2007), with positive values indexing improvements from baseline to 12, 24 or ⩾36 months follow-up. These time points were initially pooled using the last follow-up time; however, we also presented sensitivity analyses stratified by follow-up time (when at least three studies per follow-up were available). Hedges’ *g* values were interpreted as small (*g* = 0.2), medium (*g* = 0.5) or large (*g* = 0.8) effect sizes (Cohen, 1988; Hedges, 2007). Outcome e was estimated through the meta-analytical proportion [95% confidence interval (CI)] of remission in our primary outcome and OR (95% CI) in our secondary outcome.

The secondary analysis compared outcomes a–e across CHR-P individuals who developed psychosis and those who did not. Outcomes a-d were again measured with Hedge's *g*, with positive values indicating improvements in those non-transitioning compared to those transitioning. Outcome e was analysed with OR, with values greater than 1 indexing higher frequencies in those transitioning compared to those non-transitioning. Secondary analyses were conducted only when there were at least two studies per outcome comparing transitioning and non-transitioning individuals.

Because the studies were expected to be heterogeneous, meta-analytical random-effects models were used. Heterogeneity among study point estimates was assessed with the *Q* statistic. The magnitude of heterogeneity was evaluated with the *I*^2^ index. For the primary and secondary analyses, outcomes (a) to (d), publication bias was examined by visually inspecting funnel plots and applying the regression intercept of Egger for outcomes (Higgins *et al*., 2011). Publication bias is not typically assessed for proportions -outcome (e)-, as there are no undesirable or negative results that may have biased publications (Maulik *et al*., 2011); however, we tested this by conducting a meta-regression of the effect size on study's sample size. Meta-regressions were performed, when at least seven studies per outcome were available. We investigated the influence of the following factors: continent (Europe *vs.* North America *vs.* Other); type of psychometric instrument (CAARMS *vs.* SIPS *vs.* Other); quality of the study (NOS total score); CHR-P subgroups: (a) proportion of Attenuated Psychosis Symptoms (APS), (b) proportion of Brief and Limited Intermittent Psychotic Symptoms (BLIPS), (c) proportion of Genetic Risk and Deterioration syndrome (GRD), (d) proportion of Basic Symptoms (BS); mean age; sex (% female); year of publication; duration of untreated attenuated psychotic symptoms; ICD or DSM-defined comorbidity; exposure to baseline interventions. The significance level was set at *α* = 0.05, and all tests were two-tailed. Heterogeneity was considered significant when *p* < 0.10 (Fletcher, 2007). Comprehensive Meta-Analysis Software, version 3 (Biostat, Inc) was used for the analyses (Borenstein *et al*., 2013).

## Results

### Characteristics of the database

**Fig. 1.**
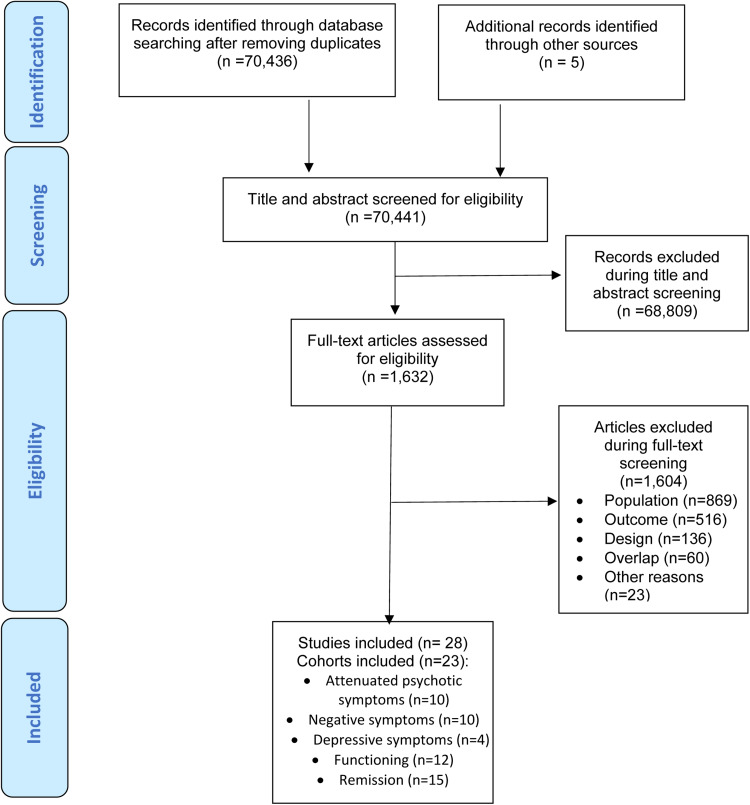
PRISMA Flowchart Outlining Study Selection Process.

The literature search yielded 70 441 citations after removing duplicates, which were screened for eligibility. Of those, 1632 were assessed for eligibility at full text. After excluding 1604 studies, 28 studies – 27 (96.4%) longitudinal cohorts and 1 (3.6%) randomised clinical trial- fulfilling our inclusion criteria from 23 cohorts were included in at least one of the meta-analysis (in descending order of frequency): 10 cohorts provided attenuated psychotic symptoms data, 10 cohorts provided negative psychotic symptoms data, four cohorts provided depressive symptoms, 12 cohorts provided functioning data, data and 15 cohorts provided remission data (Fig. 1). Of the 23 cohorts, 11 (47.8%) were conducted in Europe, six (26.1%) in North America, four (17.5%) in Asia, one (4.3%) in Australia and one (4.3%) in more than one continent. The mean duration of the follow-up of the included studies was 30.7 months (range 6–192 months). The overall database comprised 2756 CHR-P individuals (mean age = 20.4 years, 45.5% females) (Table 1, online Supplementary eTable 5).

**Table 1.** Characteristics of the included studies^a^

First author, year	Country, Continent	Design	CHR-P sample size	CHR-P subgroups	Age: mean (s.d.), range [years]	Sex: % female	CHR-P assessment tools	Total duration of follow-up [months]	NOS
Addington *et al*. (2011)	USA	Longitudinal cohort	303	N.a.	18 (4.9), 12–36	44.0	SIPS/SOPS	30	4
Addington *et al*. (2019)	Multi	Longitudinal cohort	278	N.a.	18,8 (4.4)	44.4	SIPS/SOPS	24	4
Armando *et al*. (2015)	Italy	Longitudinal cohort	35	82.9% APS, 14.3% BLIPS, 11.4% GRD	13.8 (2.1), 9–17	48.6	SIPS/SOPS	12	5
Beck *et al*. (2019*b*)	Switzerland	Longitudinal cohort	255	N.a.	24.1 (8.2), 14–57	59.0	SIPS/SOPS	192	3
Cannon *et al*. (2015)	Multi	Longitudinal cohort	274	N.a.	19.6 (4.2)	37.6	SIPS/SOPS	12	5
Chen *et al*. (2016)	China	Longitudinal cohort	63	100% APS	21.9 (4.5), 14–30	47.6	SIPS/SOPS	6	4
Cotter *et al*. (2017)	Australia	Longitudinal cohort	268	70.5% APS, 11.2% BLIPS, 24.6% GRD	18.6 (2.3), 15–30	48.9	CAARMS	178.4	5
de Wit *et al*. (2014)	Netherlands	Longitudinal cohort	44	N.a.	14.9; 2.2, 12–18	47.1	SIPS/SOPS	72	4
Falkenberg *et al*. (2017)	UK	Longitudinal cohort	34	N.a.	22.0, 14–35	29.4	CAARMS	24	4
Guo *et al*. (2019)	USA	Longitudinal cohort	117	N.a.	16.6 (3.5), 12–25	42.7	SIPS/SOPS	12	4
Kline *et al*. (2016)	USA	Longitudinal cohort	21	N.a.	16.2 (3.1), 12–22	65.0	SIPS/SOPS	6	3
Landa *et al*. (2016)	USA	Longitudinal cohort	6	66.7% APS, 16.7% BLIPS, 16.7% GRD	19.5 (1.5), 16–21	66.7	CAARMS	6.7	3
Lemos-Giráldez *et al*. (2009)	Spain	Longitudinal cohort	61	85.2% APS, 4.9% BLIPS, 9.8% GRD	21.7 (3.8), 15–31	34.4	SIPS/SOPS	36	5
Lin *et al*. (2013)	Australia	Longitudinal cohort	325	79.7% APS, 13.5% BLIPS, 28.6% GRD	19.1 (3.3), 15–30	52.9	CAARMS	178.3^b^	5
Michel *et al*. (2018)	Germany	Longitudinal cohort	194	95.9% APS, 12.9% BLIPS, 1.0% GRD, 34.5% BS	25.3 (5.9), 16.6–39.7	37.0	SIPS/SOPS	55.9^b^	4
Mittal *et al*. (2010)	USA	Longitudinal cohort	90	N.a.	15.6 (3.0), 11–29	32.2	SIPS/SOPS	24	5
Mongan *et al*. (2020)	Multi	Longitudinal cohort	133	N.a.	22.6 (4.5)	49.0	CAARMS	24	6
Pelizza *et al*. (2019)	Italy	Longitudinal cohort	55	90.9% APS, 5.5% BLIPS. 30.9% GRD	18.4 (4.9), 13–35	49.2	CAARMS, PANSS	12	6
Phillips *et al*. (2007)	Australia	Randomised clinical trial	17	N.a.	N.a., 14–30	N.a.	BPRS, SANS	48	5
Rüsch *et al*. (2015)	Switzerland	Longitudinal cohort	172	N.a.	21.4 (5.8), 13–35	41.0	SIPS/SOPS	12	3
Rutigliano *et al*. (2016)	UK	Longitudinal cohort	154	83.8% APS, 16.2% BLIPS	23.4 (4.6), 14–35	39.6	CAARMS	120	4
Ryan *et al*. (2017)	Australia	Longitudinal cohort	180	83.3% APS, 2.8% BLIPS, 23.3% GRD	18.2 (2.7), 15–24	62.8	CAARMS	12	5
Sawada *et al*. (2017)	Japan	Longitudinal cohort	47	N.a.	19.9, 3.5, 12–30	52.9	SIPS/SOPS	54	5
Shi *et al*. (2016)	China	Longitudinal cohort	32	N.a.	18.8 (1.1)	59.4	SIPS/SOPS	6	5
Velthorst *et al*. (2011)	Netherlands	Longitudinal cohort	77	N.a.	19.2 (3.8), 12–35	33.8	SIPS/SOPS	36	5
Yee *et al*. (2018)	Singapore	Longitudinal cohort	211	N.a.	21.8 (3.6), 14–29	32.4	CAARMS	24	3
Zhang *et al*. (2017)	China	Longitudinal cohort	117	78.6% APS, 3.4% BLIPS, 23.1% GRD	24.7 (7.6), 15–45	52.1	SIPS/SOPS	28.3^b^	4
Ziermans *et al*. (2011)	Netherlands	Longitudinal cohort	72	N.a.	15.3 (1.9), 12–18	38.9	SIPS/SOPS	24	4

APS, Attenuated Psychosis Symptoms; BLIPS, Brief Limited Intermittent Psychotic Symptoms; BS, Basic symptoms; CAARMS, Comprehensive Assessment of At-Risk Mental States; CHR-P, Clinical high risk of psychosis; GRD, Genetic risk and deterioration syndrome; NOS, Newcastle-Ottawa Scale; PANSS, Positive and Negative Syndrome Scale; SIPS, Structured Interview for Prodromal Syndromes.

a Overlapping samples can contribute with different outcomes.

b Mean duration of follow-up.

### Clinical outcomes within CHR-P individuals non-transitioning to psychosis

**Fig. 2.**
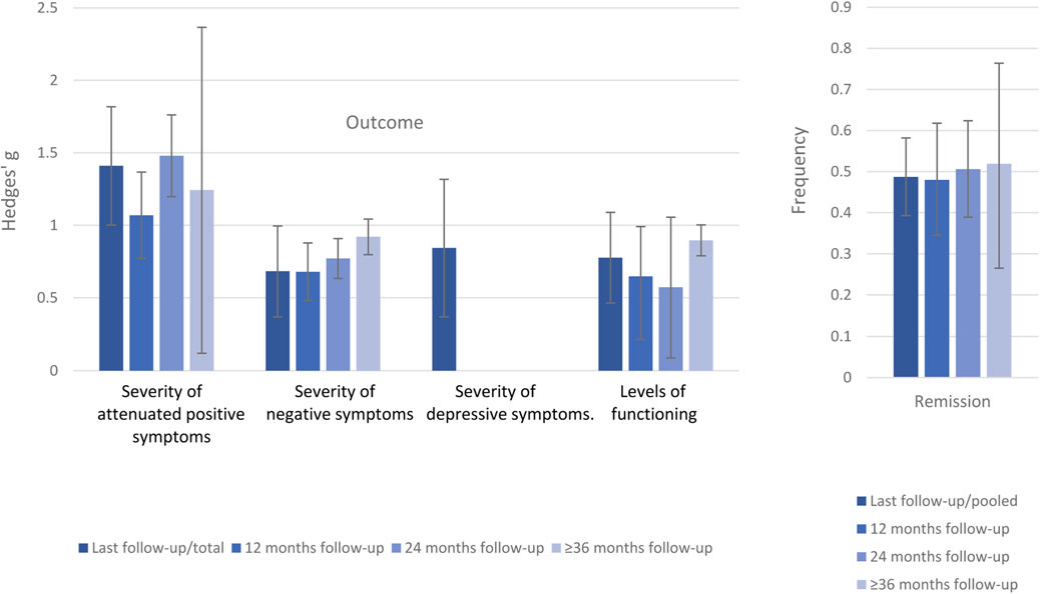
Clinical outcomes CHR-P individuals who do not transition to psychosis. Positive values of Hedge's *g* indicate improvements at follow-up compared to baseline.

Within CHR-P individuals not transitioning to psychosis, there was a baseline to follow-up improvement in attenuated psychotic symptoms (*k* = 10, *n* = 872, Hedges’ *g* = 1.410, 95% CI 1.002–1.818), negative psychotic symptoms (*k* = 10, *n* = 872, Hedges’ *g* = 0.683, 95% CI 0.371–0.995); depressive symptoms (*k* = 4, *n* = 301, Hedges’ *g* = 0.844, 95% CI 0.371–1.317) (online Supplementary eTable 6, Fig. 2) and functioning (*k* = 12, *n* = 1095, Hedges’ *g* = 0.776, 95% CI 0.463–1.089). The frequency of remission was 48.7% (95% CI 39.3–58.2%) (*k* = 15, *n* = 1219).

In the sensitivity analyses stratified by follow-up time, attenuated psychotic symptoms improved at 12- (Hedges' *g* = 1.069, 95% CI 0.772–1.367), 24- (Hedges’ *g* = 1.479, 95% CI 1.197–1.761) and ⩾36 months follow-up (Hedges’ *g* = 1.243 95% CI 0.120–2.366). Negative psychotic symptoms improved at 12- (Hedges’ *g* = 0.679, 95% CI 0.481–0.878), 24- (Hedges’ *g* = 0.771, 95% CI 0.633–0.908) and ⩾36 months follow-up (Hedges’ *g* = 0.920, 95% CI 0.797–1.043). Functioning improved at 12- (Hedges’ *g* = 0.647, 95% CI 0.393–0.991), 24- (Hedges’ *g* = 0.572, 95% CI 0.086–1.058) and ⩾36 months follow-up (Hedges’ *g* = 0.896, 95% CI 0.779–1.012). The frequency of remission was 48.0% (95% CI 34.5–61.8%) after 12 months, 50.6% (95% CI 38.9–62.4%) after 24 months and 51.9% (95% CI 26.5–76.4%) after ⩾36 months. For depressive symptoms, there were not enough data to conduct sensitivity analyses (online Supplementary eTable 6, Fig. 2).

### Clinical outcomes in CHR-P non-transitioning *v.* those transitioning to psychosis

**Fig. 3.**
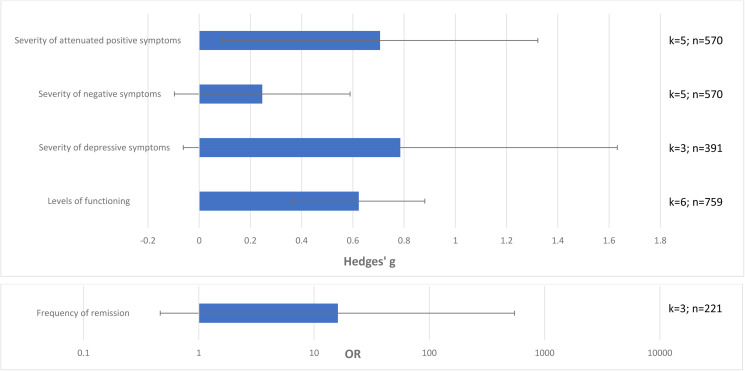
Clinical outcomes in CHR-P individuals not-transitioning to psychosis *vs.* those transitioning to psychosis at follow-up, with 95% CIs. Positive values of Hedge's *g* or OR indicate higher improvements in CHR-P individuals not transitioning to psychosis *v.* those transitioning to psychosis.

Attenuated psychotic symptoms (*k* = 5, *n* = 570, Hedges’ *g* = 0.706, 95% CI 0.091–1.322) and functioning (*k* = 6, *n* = 759, Hedges’ *g* = 0.623, 95% CI 0.375–0.871) improved in CHR-P individuals who did not transition to psychosis compared to those who transitioned to psychosis. There were no statistically significant differences in negative symptoms (*k* = 5, *n* = 570, Hedges’ *g* = 0.246, 95% CI −0.097 to 0.589), depressive symptoms (*k* = 3, *n* = 391, Hedges’ *g* = 0.785, 95% CI −0.062 to 1.632) or frequency of remission (*k* = 3, *n* = 221, OR = 16.110, 95% CI 0.473–549.02) between CHR-P individuals who did not transition to psychosis and those transitioning to psychosis (online Supplementary eTable 7, Fig. 3).

### Heterogeneity and publication bias

Heterogeneity was statistically significant for all of the primary analyses (*p* < 0.10), ranging from 79.488 (depressive symptoms) to 94.681% (functioning). Heterogeneity was also significant for all the secondary analyses, ranging from 15.823 (functioning) to 92.142% (attenuated psychotic symptoms). Egger's test was not significant for the primary analysis and secondary analyses (*p* > 0.05) (online Supplementary eTables 6 and 7).

### Quality assessment and meta-regressions

The NOS scores were 4.4 ± 0.9, ranging from 3 to 6, revealing the moderate quality of the included studies. Older age was associated with higher improvements of attenuated psychotic symptoms (*β* = 0.225, *p* = 0.012). Publication year was associated with a higher improvement of functioning (*β* = −0.124, *p* = 0.0026). Finally, a lower proportion of Brief Limited Intermittent Psychotic Symptoms was associated with higher frequencies of remission (*β* = −0.054, *p* = 0.0085). The meta-regressions did not reveal any significant association between the study continent, the psychometric instrument used, the quality of the study or the proportion of females (all *p* > 0.05) (online Supplementary eTable 8). There were not enough data to perform meta-regressions for the duration of untreated attenuated psychotic symptoms, ICD or DSM-defined comorbidity or exposure to baseline interventions.

## Discussion

To our knowledge, this is the first meta-analysis to comprehensively evaluate numerous clinical outcomes (attenuated psychotic symptoms, negative symptoms, depressive symptoms, functioning and remission) in CHR-P individuals who do not transition to psychosis. Evaluating 2756 CHR-P individuals from 23 prospective cohorts, we showed that, although CHR-P individuals improved on several outcomes, more than half of them did not reach remission.

The main finding of this meta-analysis is to have demonstrated a variable improvement of clinical outcomes over follow-up. The effect size for the improvement in the evaluated outcomes was large for attenuated psychotic symptoms (Hedges’ *g* = 1.410) and depressive symptoms (Hedges’ *g* = 0.844), and medium for negative psychotic symptoms (Hedges’ *g* = 0.683). Furthermore, there was a medium effect size for functional improvements (Hedges’ *g* = 0.776). The large effect size improvements in attenuated psychotic symptoms compared to other outcomes may be explained by a better therapeutic response for positive symptoms, which tends to respond better to antipsychotic medication or cognitive-behavioural therapy, compared to other outcomes such as negative symptoms (Woodward *et al*., 2014). However, there is no convincing evidence suggesting that specific preventive interventions can specifically improve attenuated (as opposed to established) psychotic symptoms better than others, including needs-based interventions (Davies *et al*., 2018; Fusar-Poli *et al*., 2019*a*, 2020*c*). A specific concern relates to the impact of antipsychotics in CHR-P individuals, which appears to be largely secondary to the high comorbidities accumulated and their transdiagnostic impact on the clinical presentation (Salazar de Pablo *et al*., 2020*b*; Fusar-Poli and Salazar de Pablo, 2021). Similarly, depressive symptoms seem to improve over time, which is relevant clinically given that depression and anxiety are common reasons for seeking help in CHR-P individuals (Falkenberg *et al*., 2015). Furthermore, previous meta-analytical evidence suggests that 40.7% of CHR-P individuals (Fusar-Poli *et al*., 2014) and 49% of individuals with DSM-5 Attenuated Psychosis Syndrome (Salazar de Pablo *et al*., 2019) present with comorbid depressive disorders. Previous studies have indicated that persistence of depression in CHR-P individuals is associated with decreased remission from a CHR-P status (Rutigliano *et al*., 2016; Kline *et al*., 2018; Fusar-Poli *et al*., 2019*c*). Depression is also associated with more pronounced negative psychotic symptoms and general symptoms, and it may contribute beyond the impact of positive and negative symptoms to impairments in social functioning (Fusar-Poli *et al*., 2014). Despite these findings, depressive symptoms do not lead to an increased risk of developing psychosis in CHR-P individuals (Fusar-Poli *et al*., 2014). Improvements on negative findings were less marked but still significant. We also showed that negative symptom improvements in CHR-P individuals who do not transition are of similar magnitude to those observed in individuals with schizophrenia (ES = 0.66) (Savill *et al*., 2015). Negative symptoms are often the first symptoms that CHR-P individuals develop (Metzak *et al*., 2020). There is a strong negative relationship between negative symptoms and functioning in CHR-P individuals (Devoe *et al*., 2020; Metzak *et al*., 2020), a finding also confirmed in individuals with schizophrenia (Ventura *et al*., 2009). Therefore, the observed clinical improvements in the severity of core CHR-P symptoms were paralleled by functional improvements at follow-up. It is well established that, similar to other psychiatric disorders, functional impairments are common in CHR-P individuals (Fusar-Poli *et al*., 2015*c*). However, the functional improvement may not be sufficient to reach the full functional remission (see below). Previous evidence on CHR-P individuals not transitioning to psychosis indicated that 45.3% of them still remain functionally impaired after 6 years (Rutigliano *et al*., 2016). This is not surprising given the lack of robust interventions to improve functional outcomes in this population.

Another core finding of this meta-analysis is to have complemented the analysis of continuous outcomes such as the severity of symptoms or levels of functioning with other real-world categorical outcomes that are directly informative of clinical care. In fact, statistically significant improvements of attenuated psychotic symptoms, depressive or negative symptoms and functioning do not automatically translate into tangible benefits for the lives of CHR-P individuals. This phenomenon has already been observed in psychopharmacological interventions for negative symptoms in schizophrenia, where statistically significant improvements were associated with negligible patient-level perceived improvements (Fusar-Poli *et al*., 2015*b*). Indeed, despite the symptomatic and functional improvements observed above, our meta-analytic frequency of remission indicates that only less than half (48.7%) of CHR-P not developing psychosis eventually remitted at follow-up. Our results align with a systematic review which found that 28–71% of CHR-P individuals who do not transition to psychosis, do not achieve remission (Beck *et al*., 2019*a*). Our increased frequencies of remission are due to the exclusion of CHR-P individuals who transitioned to psychosis. Interestingly, our frequency of remitters appeared lower than that observed during a first episode of psychosis (58% at 66 months) (Lally *et al*., 2017). This conflicts with one of the core foundations of the clinical staging model, which assumes that early stages are associated with a more favourable outcome and the likelihood of remission (Fusar-Poli *et al*., 2017*b*). Future research is required to address this issue.

According to our sensitivity analyses, frequencies of remission do not substantially increase throughout the follow-up. Improvements in functioning, attenuated psychotic symptoms and negative symptoms are also variable, without a significant pattern towards a prolonged improvement. This suggests that preventive interventions and monitoring may be needed in the long-term to support CHR-P individuals who do not remit, and individuals with negative symptoms and poor functioning. Currently, only 27.6% of clinical services to prevent psychosis provide care for more than 24 months (Salazar de Pablo *et al*., 2021*a*). Unfortunately, 2 years of care is not sufficient to capture the very real long-term clinical outcomes of this vulnerable population (Fusar-Poli *et al*., 2020*b*). A need for specialised services to detect CHR-P individuals and to offer needs-based and psychological interventions has been identified (Fusar-Poli *et al*., 2020*d*). This meta-analysis advances knowledge by clarifying that those CHR-P individuals who do not transition require extended support for their mental health, as non-transition does not automatically imply restoring a healthy status.

Our secondary analyses compared CHR-P individuals developing psychosis and not on the same outcomes. We found that functioning (Hedges’ *g* = 0.623) and attenuated psychotic symptoms (Hedges’ *g* = 0.706) improved in CHR-P individuals who did not transition to psychosis compared to those who did. Functioning is closely related to both the duration and severity of attenuated psychotic symptoms (Salazar de Pablo *et al*., 2020*a*). These findings indicate that the level of functioning of CHR-P individuals is strictly closed to transition to psychosis (Fusar-Poli *et al*., 2015*c*), confirming that transition to psychosis from a CHR-P state is associated with severe real-world clinical outcomes. A recent study confirmed that CHR-P individuals transitioning to psychosis (*n* = 130) were more likely to receive antipsychotic medication, to be admitted informally and on a compulsory basis, and to have spent more time in hospital than first-episode patients who presented to early intervention services (*n* = 1121), with a comparable likelihood of receiving clozapine (Fusar-Poli *et al*., 2020*a*). Surprisingly, we found no statistically significant differences in remission, negative and depressive symptoms between CHR-P individuals who transition or not to psychosis. It is important to highlight that statistical potency for these analyses was low, due to the limited number of prospective cohorts included. The lack of differences on negative or depressive features may be explained by the fact that no effective interventions are available for these domains in CHR-P individuals, beyond the presence of subthreshold or frank positive psychotic symptoms. The lack of difference in frequency of remission may be explained by the rapid and intensive package of early intervention care typically received by CHR-P individuals who develop over threshold psychosis, which has been demonstrated to be highly effective in improving clinical outcomes (Correll *et al*., 2018).

According to the meta-regressor factors tested, higher age was associated with a higher longitudinal improvement in attenuated psychotic symptoms. We previously found that age does not appear to modulate transition risk (Catalan *et al*., 2020). However, positive psychotic symptoms, such as hallucinations, are common in children and adolescents (Stevens *et al*., 2014). This may be related to the high prevalence of previous traumatic events in adolescents at CHR-P (63.2–85.0%) (Catalan *et al*., 2020), which are associated with the severity of positive symptoms, such as hallucinations and delusions (Bailey *et al*., 2018). There is also converging evidence indicating that early onset of psychosis is associated with poorer longer-term outcomes (Salazar de Pablo *et al*., 2021*b*). We also found that publication year was associated with better functioning. While declining transition risks over the years have been observed in the early days (Fusar-Poli *et al*., 2012), these have been disconfirmed in the most recent literature (Salazar de Pablo *et al*., 2021*c*). These discrepancies may be due to heterogeneous risk enrichment during the recruitment of young adults undergoing a CHR-P assessment (Fusar-Poli *et al*., 2016*c*, 2016*d*; Rice *et al*., 2019), with the associated variable impact of comorbid conditions (Salazar de Pablo *et al*., 2020*a*). Overall, the association between publication year and functioning may be secondary to sampling biases (Fusar-Poli *et al*., 2016*d*, 2018). We also found lower frequencies of remission in BLIPS individuals. This is an established finding, because BLIPS have poor mental health outcomes (Fusar-Poli *et al*., 2017*b*, 2017*d*) such as the higher risk of developing psychosis, being admitted compulsorily into hospital, receiving antipsychotics and benzodiazepines and lower probability of receiving psychotherapy (Fusar-Poli *et al*., 2016*a*, 2016*b*, 2017*a*, 2017*d*, 2019*b*, 2020*b*).

This study has several limitations. First, the available studies to meta-analyse some of the clinical outcomes (e.g., depressive and negative symptoms) evaluated was limited. As noted above, lack of power for some may have led to non-statistically significant differences in frequency of remission between CHR-P individuals who transitioned to psychosis or not. However, the database was sufficiently powered to run our primary analyses. Second, additional outcomes, such as quality of life, were not assessed as these were hardly ever reported in a meta-analysable manner. Third, there was high heterogeneity across the included studies, which we partially addressed in meta-regression analyses. Fourth, it was not possible to test some meta-regressors, including duration of untreated attenuated psychotic symptoms, ICD or DSM-defined comorbidity and exposure to baseline interventions, due to a limited amount of studies. Fifth, as noted above, the clinical meaning of changes in clinical and functional outcomes are not always directly interpretable. For instance, a decrease from 6 to 3 in one of the CHR-P symptoms may be more clinically relevant than a decrease from 3 to 0. Nevertheless, these findings confirm that transition to psychosis is not a trivial event of little clinical meaning, as argued by some authors, but related to real-world morbidity and mortality (Fusar-Poli *et al*., 2020*b*). Sixth, as transition to psychosis is defined by the worsening of attenuated psychotic symptoms, differences in secondary outcome were somewhat tautologically expected. Finally, we could not stratify our results according to the CHR-P subgroups, and we could only test their association with one of our outcomes (remission).

## Conclusion

Clinical outcomes improve in CHR-P individuals not transitioning to psychosis but only less than half remit over time. Sustained clinical attention should be provided in the longer term to monitor these outcomes.

## Data Availability

The studies included in this review were publicly available. The lead author and corresponding author can be contacted.

## Supplementary material

For supplementary material accompanying this paper visit https://doi.org/10.1017/S2045796021000639. click here to view supplementary material
