# Supplementary material for: Clinical outcomes in individuals at clinical high risk of psychosis who do not transition to psychosis: a meta-analysis
Source: Epidemiol Psychiatr Sci. 2022 Jan 19;31:e9. doi: 10.1017/S2045796021000639 (PMC8786617; doi:10.1017/S2045796021000639)
Supplement: Supplementary file 1 [file S2045796021000639sup001.docx]

**SUPPLEMENTARY MATERIAL**

**eTable 1:** PRISMA statement and checklist…..…..…..…..…..…..…....…..…..…..…....…..…..…..…..…..…..…..…..…..…...…..……….page 2-3

**eTable 2:** MOOSE checklist …..…..…...…..…..…..…....…..…..…..…...…..…..…..…..…..…..…..…..…..…..…..…..…..…...…..….........page 4-5

**eTable 3:** Definitions and instruments employed to define outcomes...…..…..…..…....…..…..…..….....…..…..…..….....…...…..…..…..page 6

**eTable 4:** Risk of bias (quality) assessment using the modified Newcastle Ottawa Scale for cohort studies …..…..………..…..………page 7

**eTable 5:** Other characteristics of the included studies………..………..…..………..…..………..…..………..…..………..…..………..….page 8-9

**eTable 6:** Outcomes in non-transitioned CHR-P individuals..…....…..…..….....…..…..…..….…..…..…..…...…..…..………..…….…......page 10

**eTable 7:** Comparison transitioned vs non-transitioned CHR-P individuals......…..…..…..…..…..…..…..…..…..…..…...…..….…..…….page 11

**eTable 8:** Moderating factors.………………………………....…..…..…..……..…..…..……..…..…..…..…....…..…..…...…….…..…..…....page 12

**eMethods 1:** CHR-P instruments included..…..…..…..…..…..…..…..…..…..…..…..…..…..…....…..…..…....…..……....…..…....……....page 13

**eMethods 2:** Data extraction details …..…..…..…..…..…..…..…..…..…..…..…..…..…..…....…..…..…....…..….......…...…....…..………page 14

**This supplementary material has been provided by the authors to give readers additional information about their work.**

**eTable 1: PRISMA statement and checklist**

| **Section/topic** | **#** | **Checklist item** | **Page** |
| --- | --- | --- | --- |
| **TITLE** | | |  |
| Title | 1 | Identify the report as a systematic review, meta-analysis, or both. | 1 |
| **ABSTRACT** | | |  |
| Structured summary | 2 | Provide a structured summary including, as applicable: background; objectives; data sources; study eligibility criteria, participants, and interventions; study appraisal and synthesis methods; results; limitations; conclusions and implications of key findings; systematic review registration number. | 2-3 |
| **INTRODUCTION** | | |  |
| Rationale | 3 | Describe the rationale for the review in the context of what is already known. | 4 |
| Objectives | 4 | Provide an explicit statement of questions being addressed with reference to participants, interventions, comparisons, outcomes, and study design (PICOS). | 4-5 |
| **METHODS** | | | |
| Protocol and registration | 5 | Indicate if a review protocol exists, if and where it can be accessed (e.g., Web address), and, if available, provide registration information including registration number. | 5 |
| Eligibility criteria | 6 | Specify study characteristics (e.g., PICOS, length of follow-up) and report characteristics (e.g., years considered, language, publication status) used as criteria for eligibility, giving rationale. | 5-6 |
| Information sources | 7 | Describe all information sources (e.g., databases with dates of coverage, contact with study authors to identify additional studies) in the search and date last searched. | 5 |
| Search | 8 | Present full electronic search strategy for at least one database, including any limits used, such that it could be repeated. | 5 |
| Study selection | 9 | State the process for selecting studies (i.e., screening, eligibility, included in systematic review, and, if applicable, included in the meta-analysis). | 5-6 |
| Data collection process | 10 | Describe method of data extraction from reports (e.g., piloted forms, independently, in duplicate) and any processes for obtaining and confirming data from investigators. | 6 |
| Data items | 11 | List and define all variables for which data were sought (e.g., PICOS, funding sources) and any assumptions and simplifications made. | 6 |
| Risk of bias in individual studies | 12 | Describe methods used for assessing risk of bias of individual studies (including specification of whether this was done at study or outcome level), and how this information is to be used in any data synthesis. | 7 |
| Summary measures | 13 | State the principal summary measures. | 7 |
| Risk of bias across studies | 15 | Specify any assessment of risk of bias (i.e. Newcastle-Ottawa Scale (NOS), that may affect the cumulative evidence. | e7 |
| Additional analyses | 16 | Describe methods of additional analyses (e.g., sensitivity or subgroup analyses, meta-regression), if done, indicating which were pre-specified. | 7-8 |
| **RESULTS**   | | | |
| Study selection | 17 | Give numbers of studies screened, assessed for eligibility, and included in the review, with reasons for exclusions at each stage, ideally with a flow diagram. | 8,  figure 1 |
| Study characteristics | 18 | For each study, present characteristics for which data were extracted (e.g., study size, PICOS, follow-up period) and provide the citations. | 24-25 |
| Risk of bias within studies | 19 | Present data on risk of bias of each study and, if available, any outcome level assessment (see item 12). | 10, 24-25 |
| Results of individual studies | 20 | For all outcomes considered (benefits or harms), present, for each study a summary data for each intervention group. | 8-9,  24-25 |
| Results synthesis | 21 | Present results of study analyzed. | 8-10, e8-10 |
| Risk of bias across studies | 22 | Present results of any assessment of risk of bias across studies (see Item 15). | 10 |
| Additional analysis | 23 | Give results of additional analyses, if done (e.g., sensitivity or subgroup analyses, meta-regression [see Item 16]). | 9-10 |
| **DISCUSSION**   | | | |
| Summary of evidence | 24 | Summarize the main findings including the strength of evidence for each main outcome; consider their relevance to key groups (e.g., healthcare providers, users, and policy makers). | 11-14 |
| Limitations | 25 | Discuss limitations at study and outcome level (e.g., risk of bias), and at review-level (e.g., incomplete retrieval of identified research, reporting bias). | 14 |
| Conclusions | 26 | Provide a general interpretation of the results in the context of other evidence, and implications for future research. | 14 |
| **FUNDING** |  |  |  |
| Funding | 27 | Describe sources of funding for the systematic review and other support (e.g., supply of data); role of funders for the systematic review. | 15 |

**eTable 2: MOOSE checklist**

| **Criteria** | | **Brief description of how the criteria were handled in the meta-analysis** |
| --- | --- | --- |
| **Reporting of background should include** | | |
| √ | Problem definition | No meta-analysis has comprehensively assessed the progression of outcomes in non-transitioned CHR-P individuals. |
| √ | Hypothesis statement | We hypothesized that outcomes would be significant in non-transitioned CHR-P individuals. |
| √ | Description of study outcomes | The outcomes are defined in eTable 3. |
| √ | Type of exposure or intervention used | We included original studies reporting outcomes after a certain follow-up period. |
| √ | Type of study designs used | Longitudinal studies only. |
| √ | Study population | CHR-P individuals according to established psychometric instruments. |
| **Reporting of search strategy should include** | | |
| √ | Qualifications of searchers | The credentials of the investigators are detailed in the manuscript. |
| √ | Search strategy, including time period included in the synthesis and keywords | Multi-step literature search detailed in methods section. |
| √ | Databases and registries searched | Pubmed and Web of Science databases. |
| √ | Use of hand searching | We carried out a manual search as specified in the manuscript. |
| √ | List of citations located and those excluded, including justifications | A PRISMA flowchart was added to the main text, including reasons for exclusion. |
| √ | Method of addressing articles published in languages other than English | Only articles in English language were included. |
| √ | Method of handling abstracts and unpublished studies | This point is detailed in the methods section. |
| √ | Description of any contact with authors | We contacted corresponding authors to request additional data when this was needed. |
| **Reporting of methods should include** | | |
| √ | Description of relevance or appropriateness of studies assembled for assessing the hypothesis  to be tested | Detailed inclusion and exclusion criteria were described in the methods section. |
| √ | Rationale for the selection and coding of data | Data was selected and extracted to answer our research questions. |
| √ | Assessment of confounding | Meta-regressions were carried out when at least 7 studies were available per outcome. |
| √ | Assessment of study quality, including blinding of quality assessors; stratification or regression on possible predictors of study results | This is detailed in the methods section and supplementary. We adapted the Newcastle-Ottawa Scale for the evaluation of cohort studies to assess the study quality. |
| √ | Assessment of heterogeneity | Heterogeneity was assessed with the I^2^ index. |
| √ | Description of statistical methods in sufficient detail to be replicated | This is detailed in the methods section. |
| √ | Provision of appropriate tables and graphics | We included several tables and graphics in the main text and supplementary section to give readers additional information about our work. |
| **Reporting of results should include** | | |
| √ | Graph summarizing individual study estimates and overall estimate | We have appended several graphs summarizing our meta-analytical estimations. |
| √ | Table giving descriptive information for each study included | We have presented descriptive information for each study in the tables and as supplementary material. |
| √ | Results of sensitivity testing | Sensitivity testing results are provided. |
| √ | Indication of statistical uncertainty of findings | We reported the 95% CI for all our estimations. |
| **Reporting of discussion should include** | | |
| √ | Quantitative assessment of bias | Quantitative assessment of bias is reported and discussed in the text. |
| √ | Justification for exclusion | Our inclusion and exclusion criteria aim to obtain the highest quality evidence possible as detailed in the manuscript. |
| √ | Assessment of quality of included studies | The quality of our studies is summarized and discussed. |
| **Reporting of conclusions should include** | | |
| √ | Consideration of alternative explanations for observed results | We discussed other explanations for our findings, specifically considering potential methodological shortcomings. |
| √ | Generalization of the conclusions | We have addressed the generalization of the conclusions in the discussion section. |
| √ | Guidelines for future research | We have suggested possible streams of future development and research in the discussion. |
| √ | Disclosure of funding source | Funding sources are detailed. No separate funding was required for this meta-analysis. |

**eTable 3:** **Definitions and instruments employed to define outcomes.**

| **Outcome** | **Definition/ Instruments Used** |
| --- | --- |
| Attenuated psychotic symptoms (change from baseline to follow-up) | Positive and Negative Syndrome Scale (PANSS) (Kay *et al.*, 1987)  Scale for the Assessment of Positive Symptoms (SAPS) (Andreasen, 1984)  Brief Psychiatric Rating Scale (BPRS) (Overall and Gorham, 1988) |
| Negative psychotic symptoms (change from baseline to follow-up) | Positive and Negative Syndrome Scale (PANSS) (Kay *et al.*, 1987)  Scale for the Assessment of Negative Symptoms (SANS) (Andreasen, 1983)  Brief Psychiatric Rating Scale (BPRS) (Overall and Gorham, 1988)  Montgomery–Åsberg Depression Rating Scale (MADRS) (Montgomery and Asberg, 1979) |
| Depressive symptoms (change from baseline to follow-up) | Hamilton Rating Scale for Depression (HAM-D) (Hamilton, 1960)  Calgary Depression Scale for Schizophrenia (CDSS) (Addington *et al.*, 1992)  Beck Depression Inventory (BDI) (Beck *et al.*, 1996) |
| Functioning (change from baseline to follow-up) | Global Assessment of Functioning (GAF) (Piersma and Boes, 1997)  Social and Occupational Functioning Assessment Scale (SOFAS ) (Morosini *et al.*, 2000)  Global Functioning: Role (GFR); Global Functioning: Social (GFS) (Niendam *et al.*, 2006; Cornblatt *et al.*, 2007) |
| Remission (% at follow-up) | Symptoms remission as defined by the psychometric instruments (e.g., SIPS/SOPS, CAARMS^a^) or CHR-P criteria remission (i.e. individuals not meeting CHR-P criteria at follow-up according to established instruments) |

^a^Definitions employed by the included individual studies: SIPS/SOPS severity <3 or ≤2 for all the attenuated positive symptoms; CAARMS total positive subscale score <5.

**eTable 4: Risk of bias (quality) assessment using the modified Newcastle Ottawa Scale for cohort studies.**

| **Criteria** | **Maximum Score** |
| --- | --- |
| Representativeness of exposed cohort (e.g. total population or random sample, selected group) | 1 |
| Method used to ascertain exposure is robust? | 1 |
| Exposed and unexposed are matched or there is an adjustment for confounding factors? | 2 |
| Assessment of outcome was blind to exposure status or used record linkage, were robust tools used? | 2 |
| Follow-up period was sufficiently long for outcomes to occur? | 1 |
| Loss to follow-up rate is reported, low (<30%), and same in exposed and non-exposed? | 1 |

**eTable 5: Other characteristics of the included studies**

| **First author, year** | **CHR-P sample size baseline** | **CHR-P sample size end of the study** | **% loss to follow-up** | **Remission definition** | **Outcomes assessed**^a^ | **% on psychotherapy** | **% on medication** |
| --- | --- | --- | --- | --- | --- | --- | --- |
| (Addington *et al.*, 2011) | 303 | 111 | 63.4 | Remission of attenuated symptoms used to index clinical high-risk status. | APS, FX, NEG | 0 | 18.1 AP |
| (Addington *et al.*, 2019) | 278 | 278 | 0 | Remission from CHR-P syndrome (i.e. scores of 2 or less on all five positive symptoms on the SIPS) | APS, FX, NEG, REM | N.a. | N.a. |
| (Armando *et al.*, 2015) | 35 | 35 | 0 | Remission from UHR status. | REM | N.a. | 0 AP; 2.9 AD; 2.9 BZ |
| (Beck *et al.*, 2019) | 255 | 72 | 71.8 | Absence of APS or BLIPS (i.e., sub-threshold severity on all positive symptom items for at least 12 consecutive months preceding latest follow-up). | FX, REM | 0 | 0 |
| (Cannon *et al.*, 2015) | 274 | 274 | 0 | N.a. | POS | N.a. | 34.7 AP |
| (Chen *et al.*, 2016) | 63 | 47 | 25.4 | N.a. | DEP, FX | N.a. | N.a. |
| (Cotter *et al.*, 2017) | 268 | 268 | 0 | N.a. | DEP | N.a. | N.a. |
| (de Wit *et al.*, 2014) | 44 | 44 | 0 | No longer exhibited positive prodromal symptoms at the sub-psychotic level. | REM, FX | N.a. | Baseline: 43.2 Any  Follow-up: 20.4 AP |
| (Falkenberg *et al.*, 2017) | 34 | 23 | 32.4 | Not fulfilling the UHR criteria. | REM | N.a. | Baseline: 8.8 AP; 20.6 AD |
| (Guo *et al.*, 2019) | 117 | 117 | 0 | All SIPS positive symptoms scores below 3. | REM | N.a. | N.a. |
| (Kline *et al.*, 2016) | 21 | 21 | 0 | Symptoms remission. | REM | N.a. | N.a. |
| (Landa *et al.*, 2016) | 6 | 6 | 0 | Significant decrease in CAARMS positive symptoms and CAARMS total global and frequency scales. | APS, DEP, FX, NEG, REM | 100 CBT | Baseline: 16.6 AP  33.3 AP+MS  16.6 AP+ANX+AD  Follow-up: 50 AP |
| (Lemos-Giráldez *et al.*, 2009) | 61 | 61 | 0 | N.a. | APS, FX, NEG | 82 CBT | 79 AP |
| (Lin *et al.*, 2013) | 325 | 325 | 0 | N.a. | DEP, FX | 8 CBT; 23.4 CT | 19.4 AP + CBT or AP + CT; 7.4 Lithium |
| (Michel *et al.*, 2018) | 246 | 246 | 0 | Remission of CHR-P according to symptomatic ultra-high risk or cognitive disturbances criteria. | FX | N.a. | Baseline: 13.8 AP; 13 AD; 0.8 MS |
| (Mittal *et al.*, 2010) | 90 | 90 | 0 | N.a. | APS, NEG | N.a. | 14.4 AP; 37.8 AD; 20 Stimulants |
| (Mongan *et al.*, 2020) | 133 | 133 | 0 | N.a. | FX | N.a. | Baseline: 27.8 AD; 11.3 AP;6.0 Hypnotics; 10.5 Other |
| (Pelizza *et al.*, 2019) | 55 | 41 | 25.5 | Not satisfying inclusion criteria for CHR-P. | REM | 4.9 | N.a. |
| (Phillips *et al.*, 2007) | 17 | 17 | 0 | N.a. | DEP, FX, NEG | N.a. | 0 |
| (Rüsch *et al.*, 2015) | 172 | 101 | 41.3 | N.a. | APS, FX, NEG | N.a. | Baseline: 19.2 AP |
| (Rutigliano *et al.*, 2016) | 154 | 74 | 51.9 | No longer presenting with APS meeting CAARMS threshold, and GAF < 60. | DEP, REM | 77.1 CBT | 4.1 APS; 25.7 APS + CBT |
| (Ryan *et al.*, 2017) | 180 | 173 | 3.9 | N.a. | APS, NEG | N.a. | N.a. |
| (Sawada *et al.*, 2017) | 47 | 39 | 17 | N.a. | APS, FX, NEG | N.a. | N.a. |
| (Shi *et al.*, 2016) | 32 | 27 | 15.6 | Remission from CHR-P. status. | DEP, REM | N.a. | N.a. |
| (Velthorst *et al.*, 2011) | 77 | 70 | 9.1 | Remission from CHR-P. status. | APS, FX, NEG | N.a. | 12.3 AP; 15.6 AD; 6.5 ANX; 6.5 MET |
| (Yee *et al.*, 2018) | 105 | 71 | 32.4 | A change in CAARMS status from positive at baseline to not meeting CHR-P. criteria at follow-up. | DEP, REM | N.a. | 0 AP; 43.8 AD; 0 MS |
| (Zhang *et al.*, 2017) | 117 | 86 | 26.5 | Positive symptoms scores of 2 or less, or the return of GAF to 90% of the previous best GAF for GRD. | REM | N.a. | 49.6 AP; 41 AD /MD; 23.9 AD/MD+APS |
| (Ziermans *et al.*, 2011) | 42 | 42 | 0 | N.a. | REM | N.a. | Baseline: 36 Any; 17 AP; 14 MS; 7 PS  2 Other; Follow-up: 38 Any; 5 AP; 14 MS; 12 PS; 0 ANX; 4 other |

AD: antidepressants; ANX: anxiolytics; AP: antipsychotics APS: attenuated psychotic symptoms; BLIPS: brief limited intermittent psychotic symptoms BZ: benzodiazepines; CAARMS: Comprehensive Assessment of At-Risk Mental States CBT: cognitive behavioural therapy; CHR-P: clinical high risk for psychosis; CT: cognitive therapy: DEP: depressive symptoms; GAF: global assessment of functioning; GRD: genetic risk and deterioration FX: functioning; MET: Methylphenidate; MS: mood stabilizers; PS: psychostimulants; NEG: negative symptoms; REM: remission SIPS: Structured Interview for Psychosis-risk Syndromes.

^a^Due to overlap some all the outcomes were not meta-analyzed.

**eTable 6: Outcomes in non-transitioned CHR-P individuals**

| **Symptom**, follow-up period | **No. of**  **Studies**^a^ | **Sample size** | **Hedges’ g** | | | **z Score** | **P** | **Test for Heterogeneity** | | | **Funnel plot assymetry** | **Egger´s test**  **p** |
| --- | --- | --- | --- | --- | --- | --- | --- | --- | --- | --- | --- | --- |
|  |  |  | **Mean** | **95 CI** | |  |  | **Q** | **I^2^** | **P** |  |  |
| **Attenuated psychotic symptoms** | | | | | | | | | | | | |
| **Last follow-up/total** | **10** | **872** | **1.410** | **1.002** | **1.818** | **6.768** | **<0.001** | **118.376** | **93.242** | **<0.001** | **N** | **0.785** |
| 12 months follow-up | 7 | 511 | 1.069 | 0.772 | 1.367 | 7.042 | <0.001 | 24.470 | 79.567 | <0.001 | N | 0.396 |
| 24 months follow-up | 4 | 455 | 1.479 | 1.197 | 1.761 | 10.287 | <0.001 | 7.085 | 71.772 | 0.029 | N | 0.779 |
| ≥36 months follow-up | 4 | 341 | 1.243 | 0.120 | 2.366 | 2.169 | 0.029 | 76.146 | 97.373 | <0.001 | N | 0.937 |
| **Negative symptoms** | | | | | | | | | | | | |
| **Last follow-up/total** | **10** | **872** | **0.683** | **0.371** | **0.995** | **4.291** | **<0.001** | **137.111** | **93.436** | **<0.001** | **N** | **0.947** |
| 12 months follow-up | 7 | 459 | 0.679 | 0.481 | 0.878 | 6.711 | <0.001 | 17.643 | 60.324 | 0.014 | N | 0.979 |
| 24 months follow-up | 4 | 503 | 0.771 | 0.633 | 0.908 | 11.003 | <0.001 | 5.749 | 30.419 | 0.219 | Y | 0.313 |
| ≥36 months follow-up | 4 | 377 | 0.920 | 0.797 | 1.043 | 14.657 | <0.001 | 31.048 | 90.337 | <0.001 | N | 0.340 |
| **Depressive symptoms** | | | | | | | | | | | | |
| **Last follow-up/total** | **4** | **301** | **0.844** | **0.371** | **1.317** | **3.495** | **<0.001** | **14.626** | **79.488** | **0.002** | **N** | **0.201** |
| **Functioning** | | | | | | | | | | | | |
| **Last follow-up/total** | **12** | **1,095** | **0.776** | **0.463** | **1.089** | **4.858** | **<0.001** | **206.805** | **94.681** | **<0.001** | **Y** | **0.134** |
| 12 months follow-up | 8 | 386 | 0.647 | 0.303 | 0.991 | 3.686 | <0.001 | 56.074 | 87.516 | <0.001 | N | 0.465 |
| 24 months follow-up | 5 | 514 | 0.572 | 0.086 | 1.058 | 2.308 | 0.021 | 74.884 | 94.658 | <0.001 | N | 0.533 |
| ≥36 months follow-up | 5 | 434 | 0.896 | 0.779 | 1.012 | 15.077 | <0.001 | 74.289 | 94.616 | <0.001 | N | 0.232 |
|  | | | | | | | | | | | | |
| **Symptom**, follow-up period | **No. of**  **Studies**^a^ | **Sample size** | **Proportion** | | | **z Score** | **P** | **Test for Heterogeneity** | | | **Publication bias asessment^b^** | |
|  |  |  | **%** | **95 CI** | |  |  | **Q** | **I^2^** | **P** |  |  |
| **Remission** | | | | | | | | | | | | |
| **Last follow-up/total** | **15** | **1,219** | **0.487** | **0.393** | **0.582** | **-0.260** | **0.795** | **117.236** | **88.058** | **<0.001** | β=-0.003, p=0.253 | |
| 12 months follow-up | 6 | 240 | 0.480 | 0.345 | 0.618 | -0.285 | 0.776 | 15.643 | 68.037 | 0.008 |  |  |
| 24 months follow-up | 5 | 534 | 0.506 | 0.389 | 0.624 | 0.105 | 0.916 | 22.229 | 82.006 | <0.001 |  |  |
| ≥36 months follow-up | 5 | 464 | 0.519 | 0.265 | 0.764 | 0.137 | 0.891 | 81.484 | 95.091 | <0.001 |  |  |

^a^Overlapping samples can contribute with different outcomes; ^b^Metaregression of the effect size on study’s sample size.

**eTable 7: Comparison transitioned vs non-transitioned CHR-P individuals**

|  | | | | | | | | | | | | | |
| --- | --- | --- | --- | --- | --- | --- | --- | --- | --- | --- | --- | --- | --- |
| **Symptom**, follow-up period | **No. of**  **Studies**^a^ | **Sample size no transition** | **Sample size transition** | **Hedges’ g** | | | **z Score** | **P** | **Test for Heterogeneity** | | | **Funnel plot assymetry** | **Egger´s**  **test**  **p** |
|  |  |  |  | **%** | **95 CI** | |  |  | **Q** | **I^2^** | **P** |  |  |
| **Attenuated psychotic symptoms** | 5 | 405 | 165 | 0.706 | 0.091 | 1.322 | 2.249 | 0.025 | 38.178 | 92.142 | <0.001 | N | 0.762 |
| **Negative symptoms** | 5 | 405 | 165 | 0.246 | -0.097 | 0.589 | 1.407 | 0.159 | 15.163 | 73.619 | 0.004 | N | 0.202 |
| **Depressive symptoms** | 3 | 295 | 96 | 0.785 | -0.062 | 1.632 | 1.817 | 0.069 | 9.800 | 79.591 | 0.007 | N | 0.363 |
| **Functioning** | 6 | 545 | 214 | 0.623 | 0.375 | 0.871 | 4.925 | <0.001 | 68.400 | 15.823 | 0.007 | N | 0.465 |
|  | | | | | | | | | | | | | |
| **Symptom**, follow-up period | **No. of**  **Studies**^a^ | **Sample size no transition** | **Sample size transition** | **Proportion** | | | **z Score** | **P** | **Test for Heterogeneity** | | | **Publication bias asessment^b^** | |
|  |  |  |  | **OR** | **95 CI** | |  |  | **Q** | **I^2^** | **P** |  |  |
| **Remission** | 3 | 148 | 73 | 16.110 | 0.473 | 549.02 | 1.544 | 0.123 | 15.836 | 87.371 | <0.001 | β=0.037, p=0.252 | |

*Trim and fill method was applied and small effect bias was not identified.

^a^Overlapping samples can contribute with different outcomes; ^b^Metaregression of the effect size on study’s sample size.

**eTable 8: Moderating factors**

| **Outcome** | **Meta-regressor**^a^ | **No. of Studies** | **β Coefficient** | **SE** | **95% CI** | | | **Z-Value** | **P value** |
| --- | --- | --- | --- | --- | --- | --- | --- | --- | --- |
| **Attenuated psychotic symptoms** | Continent | 10 | 0.542 | 0.765 | -0.957 | 2.042 | 0.709 | | 0.478 |
|  | Psychometric instrument | 10 | -0.529 | 0.663 | -1.829 | 0.770 | -0.798 | | 0.425 |
|  | Quality of the study | 10 | -0.029 | 0.421 | -0.855 | 0.797 | -0.068 | | 0.946 |
|  | Mean age | 10 | 0.225 | 0.090 | 0.048 | 0.402 | 2.499 | | **0.012** |
|  | Sex | 10 | -0.021 | 0.020 | -0.060 | 0.018 | -1.057 | | 0.290 |
|  | Year of publication | 10 | -0.0959 | 0.071 | -0.236 | 0.044 | -1.344 | | 0.179 |
|  | Follow-up period | 10 | -0.0043 | 0.005 | -0.014 | 0.0057 | -0.838 | | 0.402 |
| **Negative symptoms** | Continent | 10 | 0.553 | 0.956 | -1.322 | 2.427 | 0.578 | | 0.563 |
|  | Psychometric instrument | 10 | -0.120 | 0.465 | -1.031 | 0.791 | -0.259 | | 0.796 |
|  | Quality of the study | 10 | 0.261 | 0.192 | -0.115 | 0.064 | 1.359 | | 0.174 |
|  | Mean age | 10 | 0.072 | 0.092 | -0.109 | 0.253 | 0.780 | | 0.435 |
|  | Sex | 10 | -0.0046 | 0.018 | -0.040 | 0.031 | -0.250 | | 0.803 |
|  | Year of publication | 10 | -0.092 | 0.053 | -0.196 | 0.012 | -1.738 | | 0.082 |
|  | Follow-up period | 10 | 0.00037 | 0.0035 | -0.0065 | 0.0073 | 0.105 | | 0.916 |
| **Functioning** | Continent | 12 | 0.259 | 0.920 | -1.545 | 2.062 | 0.281 | | 0.779 |
|  | Psychometric instrument | 12 | 0.397 | 0.422 | -0.430 | 1.223 | 0.941 | | 0.347 |
|  | Quality of the study | 12 | -0.028 | 0.188 | -0.396 | 0.340 | -0.150 | | 0.881 |
|  | Mean age | 12 | 0.058 | 0.071 | -0.081 | 0.197 | 0.815 | | 0.415 |
|  | Sex | 12 | -0.019 | 0.021 | -0.059 | 0.022 | -0.911 | | 0.362 |
|  | Year of publication | 12 | -0.124 | 0.041 | -0.204 | -0.043 | -3.013 | | **0.0026** |
|  | Follow-up period | 12 | 0.0029 | 0.0027 | -0.0023 | 0.0081 | 1.097 | | 0.273 |
| **Remission** | Continent | 15 | -0.714 | 1.110 | -2.888 | 1.460 | -0.643 | | 0.520 |
|  | Psychometric instrument | 15 | 0.889 | 0.581 | -0.248 | 2.027 | 1.532 | | 0.126 |
|  | Quality of the study | 15 | -0.144 | 0.437 | -1.000 | 0.711 | -0.331 | | 0.741 |
|  | APS | 7 | -0.009 | 0.005 | -0.019 | 0.0015 | -1.67 | | 0.094 |
|  | BLIPS | 7 | -0.054 | 0.021 | -0.094 | -0.014 | -2.633 | | **0.0085** |
|  | GRD | 7 | -0.0034 | 0.016 | -0.034 | 0.027 | -0.217 | | 0.828 |
|  | Mean age | 15 | 0.027 | 0.086 | -0.142 | 0.195 | 0.312 | | 0.755 |
|  | Sex | 15 | 0.087 | 0.051 | -0.012 | 0.187 | 1.719 | | 0.086 |
|  | Year of publication | 15 | -0.014 | 0.140 | -0.288 | 0.260 | -0.098 | | 0.922 |
|  | Follow-up period | 15 | 0.00045 | 0.0033 | -0.0061 | 0.0070 | 0.134 | | 0.893 |

^a^Some meta-regressors could not be analysed due to limited amount of studies.

APS: Attenuated Psychosis Symptoms; BLIPS: Brief and Limited Intermittent Psychotic Symptoms; GRD: Genetic Risk and Deterioration.

**eMethods 1** Types of CHR-P assessments included (modified from (Fusar-Poli *et al.*, 2020))

The CHR-P state comprises the Ultra High Risk state and/or the Basic Symptoms (Fusar-Poli *et al.*, 2020).

- The following UHR instruments were considered to define the UHR state: Comprehensive Assessment of At-Risk Mental States (CAARMS) (Yung *et al.*, 2005) and Structured Interview for Psychosis-risk Syndromes (SIPS) (Fusar-Poli *et al.*, 2016; McGlashan T, 2010) and Early Recognition Inventory (ERIraos) (Haefner *et al.*, 2011). Furthermore, before the development of these instruments, the CHR-P state was defined through the Positive and Negative Syndrome Scale (PANSS) (Kay *et al.*, 1987), Brief Psychiatric Rating Scale (BPRS) (Overall and Gorham, 1988).
- The following UHR instruments were considered to define the BS (Fusar-Poli *et al.*, 2020): Bonn Scale for the Assessment of Basic Symptoms (BSABS) (Vollmer-Larsen *et al.*, 2007), Basel Screening Instrument for Psychosis (BSIP) (Riecher-Rössler *et al.*, 2008), and Schizophrenia Proneness Instrument (Fux *et al.*, 2013) - Adult (SPI-A) and Child and Youth (SPI-CY) version -.
- Transition to psychosis was operationalised as defined by each CHR-P instruments or according to ICD/DSM criteria.
- Basic symptoms are subjectively experienced disturbances in thought, affect, motor functioning, bodily sensation, perception and tolerance of stress (Schultze-Lutter and Theodoridou, 2017).

**eMethods 2: Data extraction details**

1. **Main characteristics of the included studies:**

- First author and year of publication
- Country
- Design (longitudinal cohort, non-randomized clinical trial, randomized clinical trial)
- CHR-P sample size
- CHR-P subgroups (% Attenuated Psychosis Symptoms -APS-, % Brief Limited Intermittent Psychotic Symptoms -BLIPS-,% Genetic risk and deterioration syndrome -GRD- and % Basic symptoms -BS-);
- Age (mean, SD, range)
- Sex (% female);
- CHR-P assessment tools (as listed in eMethods 1);
- Follow up period (in months);

1. **Main outcomes:**

- Attenuated psychotic symptoms: PANSS, SAPS, BPRS (mean±SD); at baseline and follow-up
- Negative symptoms: PANSS, SANS, BPRS (mean±SD); at baseline and follow-up
- Depressive symptoms: MADRS, HAM-D, CDSS, BDI (mean±SD); at baseline and follow-up
- Functioning GAF, SOFAS, GFS (mean±SD); at baseline and follow-up
- Remission %; at follow-up

1. **Information to detect overlapping studies:**
   - Study program, recruitment period (if applicable)
   - City, country
2. **Meta-regression analyses**

- Continent (Europe vs North America vs Other)
- Psychometric instrument (CAARMS, vs SIPS vs other)
- Quality of the study
- Proportion of Attenuated Psychosis Symptoms -APS-
- Proportion of Brief Limited Intermittent Psychotic Symptoms -BLIPS-
- Proportion of Genetic risk and deterioration syndrome -GRD-
- Proportion of Basic symptoms -BS-
- Age (mean age)
- Sex (% female)
- Year of publication
- Follow-up period
- Duration of untreated attenuated psychotic symptoms – in months- (as per (Fusar-Poli *et al.*, 2012))
- ICD or DSM-defined comorbidity: a) any non-psychotic mental disorder; b) any mood disorder c) major depressive disorder; d) depressive disorders; e) bipolar disorder type I; f) other bipolar disorders; g) personality disorders; h) borderline personality disorder; i) neurodevelopmental disorders; j) autism spectrum disorders; k) anxiety disorders; l) ADHD; m) cannabis use disorder; n) alcohol use disorder; o) stimulant use disorder; p) other substance use disorder; q) PTSD; r) OCD
- Exposure to baseline interventions: a) antipsychotics b) antidepressants c) other psychotropics d) psychotherapy [including CBT, IPT and other psychotherapeutic interventions].

**REFERENCES**

**Addington D, Addington J, Maticka-Tyndale E and Joyce J**. (1992) Reliability and validity of a depression rating scale for schizophrenics. *Schizophrenia Research* **6**: 201-208.

**Addington J, Cornblatt BA, Cadenhead KS, Cannon TD, McGlashan TH, Perkins DO, Seidman LJ, Tsuang MT, Walker EF, Woods SW and Heinssen R**. (2011) At clinical high risk for psychosis: outcome for nonconverters. *The American Journal of Psychiatry* **168**: 800-805.

**Addington J, Stowkowy J, Liu L, Cadenhead KS, Cannon TD, Cornblatt BA, McGlashan TH, Perkins DO, Seidman LJ, Tsuang MT, Walker EF, Bearden CE, Mathalon DH, Santesteban-Echarri O and Woods SW**. (2019) Clinical and functional characteristics of youth at clinical high-risk for psychosis who do not transition to psychosis. *Psychological Medicine* **49**: 1670-1677.

**Andreasen N**. (1983) Scale for the Assessment of Negative Symptoms (SANS). Iowa City: University of Iowa.

**Andreasen N**. (1984) Scale for the Assessment of Positive Symptoms (SAPS). Iowa City: University of Iowa.

**Armando M, Pontillo M, De Crescenzo F, Mazzone L, Monducci E, Lo Cascio N, Santonastaso O, Pucciarini ML, Vicari S, Schimmelmann BG and Schultze-Lutter F**. (2015) Twelve-month psychosis-predictive value of the ultra-high risk criteria in children and adolescents. *Schizophrenia Research* **169**: 186-192.

**Beck A, Steer R and Brown G**. (1996) Manual for the beck depression inventory-II. Psychological Corporation, San Antonio, TX.

**Beck K, Andreou C, Studerus E, Heitz U, Ittig S, Leanza L and Riecher-Rössler A**. (2019) Clinical and functional long-term outcome of patients at clinical high risk (CHR) for psychosis without transition to psychosis: A systematic review. *Schizophrenia Research* **210**: 39-47.

**Cannon TD, Chung Y, He G, Sun D, Jacobson A, van Erp TG, McEwen S, Addington J, Bearden CE, Cadenhead K, Cornblatt B, Mathalon DH, McGlashan T, Perkins D, Jeffries C, Seidman LJ, Tsuang M, Walker E, Woods SW, Heinssen R and Consortium NAPLS**. (2015) Progressive reduction in cortical thickness as psychosis develops: a multisite longitudinal neuroimaging study of youth at elevated clinical risk. *Biological Psychiatry* **77**: 147-157.

**Chen FZ, Wang Y, Sun XR, Yao YH, Zhang N, Qiao HF, Zhang L, Li ZJ, Lin H, Lu Z, Li J, Chan RCK and Zhao XD**. (2016) Emotional Experiences Predict the Conversion of Individuals with Attenuated Psychosis Syndrome to Psychosis: A 6-Month Follow up Study. *Frontiers in Psychology* **7**.

**Cornblatt BA, Auther AM, Niendam T, Smith CW, Zinberg J, Bearden CE and Cannon TD**. (2007) Preliminary findings for two new measures of social and role functioning in the prodromal phase of schizophrenia. *Schizophrenia Bulletin* **33**: 688-702.

**Cotter J, Lin A, Drake RJ, Thompson A, Nelson B, McGorry P, Wood SJ and Yung AR**. (2017) Long-term employment among people at ultra-high risk for psychosis. *Schizophrenia Research* **184**: 26-31.

**de Wit S, Schothorst PF, Oranje B, Ziermans TB, Durston S and Kahn RS**. (2014) Adolescents at ultra-high risk for psychosis: long-term outcome of individuals who recover from their at-risk state. *European Neuropsychopharmacology* **24**: 865-873.

**Falkenberg I, Valli I, Raffin M, Broome MR, Fusar-Poli P, Matthiasson P, Picchioni M and McGuire P**. (2017) Pattern of activation during delayed matching to sample task predicts functional outcome in people at ultra high risk for psychosis. *Schizophrenia Research* **181**: 86-93.

**Fusar-Poli P, Bonoldi I, Yung AR, Borgwardt S, Kempton MJ, Valmaggia L, Barale F, Caverzasi E and McGuire P**. (2012) Predicting psychosis: meta-analysis of transition outcomes in individuals at high clinical risk. *Archives Of General Psychiatry* **69**: 220-229.

**Fusar-Poli P, Cappucciati M, Rutigliano G, Lee TY, Beverly Q, Bonoldi I, Lelli J, Kaar SJ, Gago E, Rocchetti M, Patel R, Bhavsar V, Tognin S, Badger S, Calem M, Lim K, Kwon JS, Perez J and McGuire P**. (2016) Towards a Standard Psychometric Diagnostic Interview for Subjects at Ultra High Risk of Psychosis: CAARMS versus SIPS. *Psychiatry Journal* **2016**: 7146341.

**Fusar-Poli P, Salazar de Pablo G, Correll CU, Meyer-Lindenberg A, Millan MJ, Borgwardt S, Galderisi S, Bechdolf A, Pfennig A, Kessing LV, van Amelsvoort T, Nieman DH, Domschke K, Krebs MO, Koutsouleris N, McGuire P, Do KQ and Arango C**. (2020) Prevention of Psychosis: Advances in Detection, Prognosis, and Intervention. *JAMA Psychiatry*.

**Fux L, Walger P, Schimmelmann BG and Schultze-Lutter F**. (2013) The Schizophrenia Proneness Instrument, Child and Youth version (SPI-CY): practicability and discriminative validity. *Schizophrenia Research* **146**: 69-78.

**Guo JY, Niendam TA, Auther AM, Carrion RE, Cornblatt BA, Ragland JD, Adelsheim S, Calkins R, Sale TG, Taylor SF, McFarlane WR and Carter CS**. (2019) Predicting psychosis risk using a specific measure of cognitive control: a 12-month longitudinal study. *Psychological Medicine*: 1-10.

**Haefner H, Bechdolf A, Klosterkotter J and Maurer K**. (2011) Early detection and intervention in psychosis. A practice handbook. Stuttgart: Schattauer.

**Hamilton M**. (1960) A rating scale for depression. *Journal of Neurology Neurosurgery and Psychiatry* **23**: 56-62.

**Kay SR, Fiszbein A and Opler LA**. (1987) The positive and negative syndrome scale (PANSS) for schizophrenia. *Schizophrenia Bulletin* **13**: 261-276.

**Kline E, Thompson E, Demro C, Bussell K, Reeves G and Schiffman J**. (2016) Self-Report Instruments for Clinical Monitoring of Psychosis Risk States. *Psychiatric Services* **67**: 456-459.

**Landa Y, Mueser KT, Wyka KE, Shreck E, Jespersen R, Jacobs MA, Griffin KW, van der Gaag M, Reyna VF, Beck AT, Silbersweig DA and Walkup JT**. (2016) Development of a group and family-based cognitive behavioural therapy program for youth at risk for psychosis. *Early Intervention in Psychiatry* **10**: 511-521.

**Lemos-Giráldez S, Vallina-Fernández O, Fernández-Iglesias P, Vallejo-Seco G, Fonseca-Pedrero E, Paíno-Piñeiro M, Sierra-Baigrie S, García-Pelayo P, Pedrejón-Molino C, Alonso-Bada S, Gutiérrez-Pérez A and Ortega-Ferrández JA**. (2009) Symptomatic and functional outcome in youth at ultra-high risk for psychosis: a longitudinal study. *Schizophrenia Research* **115**: 121-129.

**Lin A, Yung AR, Nelson B, Brewer WJ, Riley R, Simmons M, Pantelis C and Wood SJ**. (2013) Neurocognitive predictors of transition to psychosis: medium- to long-term findings from a sample at ultra-high risk for psychosis. *Psychological Medicine* **43**: 2349-2360.

**McGlashan T WB, Woods S.** (2010) *The psychosis-risk syndrome: handbook for diagnosis and follow-up.*: Oxford: Oxford University

**Michel C, Ruhrmann S, Schimmelmann BG, Klosterkötter J and Schultze-Lutter F**. (2018) Course of clinical high-risk states for psychosis beyond conversion. *European Archives of Psychiatry and Clinical Neuroscience* **268**: 39-48.

**Mittal VA, Walker EF, Bearden CE, Walder D, Trottman H, Daley M, Simone A and Cannon TD**. (2010) Markers of basal ganglia dysfunction and conversion to psychosis: neurocognitive deficits and dyskinesias in the prodromal period. *Biological Psychiatry* **68**: 93-99.

**Mongan D, Föcking M, Healy C, Susai SR, Heurich M, Wynne K, Nelson B, McGorry PD, Amminger GP, Nordentoft M, Krebs MO, Riecher-Rössler A, Bressan RA, Barrantes-Vidal N, Borgwardt S, Ruhrmann S, Sachs G, Pantelis C, van der Gaag M, de Haan L, Valmaggia L, Pollak TA, Kempton MJ, Rutten BPF, Whelan R, Cannon M, Zammit S, Cagney G, Cotter DR, McGuire P and Group ENoNSNSG-EIE-GHRS**. (2020) Development of Proteomic Prediction Models for Transition to Psychotic Disorder in the Clinical High-Risk State and Psychotic Experiences in Adolescence. *JAMA Psychiatry*.

**Montgomery SA and Asberg M**. (1979) A new depression scale designed to be sensitive to change. *British Journal of Psychiatry* **134**: 382-389.

**Morosini PL, Magliano L, Brambilla L, Ugolini S and Pioli R**. (2000) Development, reliability and acceptability of a new version of the DSM-IV Social and Occupational Functioning Assessment Scale (SOFAS) to assess routine social functioning. *Acta Psychiatrica Scandinavica* **101**: 323-329.

**Niendam TA, Bearden CE, Johnson JK, McKinley M, Loewy R, O'Brien M, Nuechterlein KH, Green MF and Cannon TD**. (2006) Neurocognitive performance and functional disability in the psychosis prodrome. *Schizophrenia Research* **84**: 100-111.

**Overall J and Gorham D**. (1988) The Brief Psychiatric Rating Scale (BPRS): recent developments in ascertainment and scaling. *Psychopharmacology Bulletin* **24**: 97-99.

**Pelizza L, Paterlini F, Azzali S, Garlassi S, Scazza I, Pupo S, Simmons M, Nelson B and Raballo A**. (2019) The approved Italian version of the comprehensive assessment of at-risk mental states (CAARMS-ITA): Field test and psychometric features. *Early Intervention in Psychiatry* **13**: 810-817.

**Phillips LJ, McGorry PD, Yuen HP, Ward J, Donovan K, Kelly D, Francey SM and Yung AR**. (2007) Medium term follow-up of a randomized controlled trial of interventions for young people at ultra high risk of psychosis. *Schizophrenia Research* **96**: 25-33.

**Piersma HL and Boes JL**. (1997) The GAF and psychiatric outcome: a descriptive report. *Community Mental Health Journal* **33**: 35-41.

**Riecher-Rössler A, Aston J, Ventura J, Merlo M, Borgwardt S, Gschwandtner U and Stieglitz RD**. (2008) [The Basel Screening Instrument for Psychosis (BSIP): development, structure, reliability and validity]. *Fortschritte der Neurologie-Psychiatrie* **76**: 207-216.

**Rüsch N, Heekeren K, Theodoridou A, Müller M, Corrigan PW, Mayer B, Metzler S, Dvorsky D, Walitza S and Rössler W**. (2015) Stigma as a stressor and transition to schizophrenia after one year among young people at risk of psychosis. *Schizophrenia Research* **166**: 43-48.

**Rutigliano G, Valmaggia L, Landi P, Frascarelli M, Cappucciati M, Sear V, Rocchetti M, De Micheli A, Jones C, Palombini E, McGuire P and Fusar-Poli P**. (2016) Persistence or recurrence of non-psychotic comorbid mental disorders associated with 6-year poor functional outcomes in patients at ultra high risk for psychosis. *Journal of Affective Disorders* **203**: 101-110.

**Ryan J, Graham A, Nelson B and Yung A**. (2017) Borderline personality pathology in young people at ultra high risk of developing a psychotic disorder. *Early Intervention in Psychiatry* **11**: 208-214.

**Sawada K, Kanehara A, Sakakibara E, Eguchi S, Tada M, Satomura Y, Suga M, Koike S and Kasai K**. (2017) Identifying neurocognitive markers for outcome prediction of global functioning in individuals with first-episode and ultra-high-risk for psychosis. *Psychiatry and Clinical Neurosciences* **71**: 318-327.

**Schultze-Lutter F and Theodoridou A**. (2017) The concept of basic symptoms: its scientific and clinical relevance. *World Journal of Psychiatry* **16**: 104-105.

**Shi J, Wang L, Yao Y, Chen F, Su N, Zhao X and Zhan C**. (2016) Protective factors in Chinese university students at clinical high risk for psychosis. *Psychiatry Research* **239**: 239-244.

**Velthorst E, Nieman DH, Klaassen RM, Becker HE, Dingemans PM, Linszen DH and De Haan L**. (2011) Three-year course of clinical symptomatology in young people at ultra high risk for transition to psychosis. *Acta Psychiatrica Scandinavica* **123**: 36-42.

**Vollmer-Larsen A, Handest P and Parnas J**. (2007) Reliability of measuring anomalous experience: the Bonn Scale for the Assessment of Basic Symptoms. *Psychopathology* **40**: 345-348.

**Yee JY, Lee T-S and Lee J**. (2018) Levels of Serum Brain-Derived Neurotropic Factor in Individuals at Ultra-High Risk for Psychosis-Findings from the Longitudinal Youth at Risk Study (LYRIKS). *International Journal of Neuropsychopharmacology* **21**: 734-739.

**Yung AR, Yuen HP, McGorry PD, Phillips LJ, Kelly D, Dell'Olio M, Francey SM, Cosgrave EM, Killackey E, Stanford C, Godfrey K and Buckby J**. (2005) Mapping the onset of psychosis: the Comprehensive Assessment of At-Risk Mental States. *Australian and New Zealand Journal of Psychiatry* **39**: 964-971.

**Zhang TH, Li HJ, Woodberry KA, Xu LH, Tang YY, Guo Q, Cui HR, Liu XH, Chow A, Li CB, Jiang KD, Xiao ZP, Seidman LJ and Wang JJ**. (2017) Two-year follow-up of a Chinese sample at clinical high risk for psychosis: timeline of symptoms, help-seeking and conversion. *Epidemiology and Psychiatric Sciences* **26**: 287-298.

**Ziermans TB, Schothorst PF, Sprong M and van Engeland H**. (2011) Transition and remission in adolescents at ultra-high risk for psychosis. *Schizophrenia Research* **126**: 58-64.
